# Molecular Evolution of Ultraspiracle Protein (USP/RXR) in Insects

**DOI:** 10.1371/journal.pone.0023416

**Published:** 2011-08-25

**Authors:** Ekaterina F. Hult, Stephen S. Tobe, Belinda S. W. Chang

**Affiliations:** 1 Department of Cell and Systems Biology, University of Toronto, Toronto, Ontario, Canada; 2 Department of Ecology and Evolutionary Biology, University of Toronto, Toronto, Ontario, Canada; 3 Centre for the Analysis of Genome Evolution and Function, University of Toronto, Toronto, Ontario, Canada; California State University Fullerton, United States of America

## Abstract

Ultraspiracle protein/retinoid X receptor (USP/RXR) is a nuclear receptor and transcription factor which is an essential component of a heterodimeric receptor complex with the ecdysone receptor (EcR). In insects this complex binds ecdysteroids and plays an important role in the regulation of growth, development, metamorphosis and reproduction. In some holometabolous insects, including Lepidoptera and Diptera, USP/RXR is thought to have experienced several important shifts in function. These include the acquisition of novel ligand-binding properties and an expanded dimerization interface with EcR. In light of these recent hypotheses, we implemented codon-based likelihood methods to investigate if the proposed shifts in function are reflected in changes in site-specific evolutionary rates across functional and structural motifs in insect USP/RXR sequences, and if there is any evidence for positive selection at functionally important sites. Our results reveal evidence of positive selection acting on sites within the loop connecting helices H1 and H3, the ligand-binding pocket, and the dimer interface in the holometabolous lineage leading to the Lepidoptera/Diptera/Trichoptera. Similar analyses conducted using EcR sequences did not indicate positive selection. However, analyses allowing for variation across sites demonstrated elevated non-synonymous/synonymous rate ratios (*d*_N_/*d*_S_), suggesting relaxed constraint, within the dimerization interface of both USP/RXR and EcR as well as within the coactivator binding groove and helix H12 of USP/RXR. Since the above methods are based on the assumption that *d*_S_ is constant among sites, we also used more recent models which relax this assumption and obtained results consistent with traditional random-sites models. Overall our findings support the evolution of novel function in USP/RXR of more derived holometabolous insects, and are consistent with shifts in structure and function which may have increased USP/RXR reliance on EcR for cofactor recruitment. Moreover, these findings raise important questions regarding hypotheses which suggest the independent activation of USP/RXR by its own ligand.

## Introduction

USP/RXR, a group II nuclear receptor and transcription factor which belongs to the steroid receptor superfamily, is found across a diversity of Metazoan species ranging from sponges to mammals [1]. First characterized in *Drosophila melanogaster*, USP is the insect homolog of vertebrate RXR [2], [3]. In insects USP/RXR is involved in an array of functions including metamorphosis, reproduction, growth and development (see [4], [5], [6], [7], [8] for examples). USP/RXR is known primarily as the partner of the ecdysone receptor (EcR). In response to the binding of 20-hydroxyecdysone (20E) to EcR, heterodimerization between USP/RXR and EcR occurs and the complex then binds DNA to transactivate genes [9], [10], [11]. However, it is the controversy over whether or not USP/RXR binds juvenile hormone (JH) in insects, and is activated independent of EcR, which has led to the most debate regarding the function and evolution this receptor [12], [13], [14].

In vertebrates, 9-*cis* retinoic acid (9cRA) was identified as the high affinity ligand of the RXR receptor [15], [16], [17]. However, in insects no natural ligand has been conclusively identified, and USP/RXR remains an orphan receptor. JH has been suggested as a candidate ligand [12], but only experimental evidence from the dipteran *D. melanogaster* supports this hypothesis. In cell lines expressing *Drosophila* USP, the application of JH III induces the transcription of a transfected promoter, suggesting that JH binds USP resulting in a functional outcome [13], [18], [19]. Fluorescence-binding assays have shown that *Drosophila* USP binds not only JH III but also the JH precursors farnesol, farnesoic acid, and methyl farnesoate [20]. However, JH does not appear to directly bind with USP/RXR in less derived insects such as the holometabolous *Tribolium castaneum* or the hemimetabolous *Locusta migratoria* [14], [21]. There is also little evidence to suggest a high affinity for retinoids in insects [2], [21]. However, recent displacement binding experiments have shown that USP/RXR may bind 9cRA and all-*trans* RA at the high nanomolar range in *L. migratoria* [22]. Given the small quantity of RA found in *Locusta*, the physiological significance of this *in vitro* finding is unclear.

Lineage specific variation is also evident in the structure of USP/RXR. A comparative analysis of structural data demonstrates key differences in the ligand-binding pocket (LBP) of USP/RXR in insects. Crystallography data have shown that Dipteran and Lepidopteran USP share a conserved ligand-binding domain (LBD) with a large hydrophobic cavity capable of accepting a natural ligand [23], [24], [25]. In both the Diptera and Lepidoptera, USP copurified from the bacterial expression system with a phospholipid occupying the LBP. However, the identity of the endogenous ligand is unknown. In contrast, crystal structures of USP/RXR from two less derived insects, the red flour beetle *T. castaneum* and the sweet potato whitefly *Bemisia tabaci*, reveal a collapsed LBP [21], [26]. Given the taxonomic relationships of insects from which USP/RXR has been crystallized, this data implies a derived open pocket in higher insects. Despite the open LBP, Dipteran and Lepidopteran USP may not function independently as ligand-dependent transcription factors. In these species the ligand-dependent activation function (AF-2) domain in α-helix H12, is locked in an antagonistic conformation [23], [24], [25]. As a consequence of interactions between H3, H11–H12 and a highly conserved insertion in the loop connecting helices H1 and H3 (L1–3), residues in H12 occupy the coactivator groove (H3, loop L3–4 and H4), precluding the agonist conformation and preventing the binding of transcriptional coactivators [23], [24], [27].

These striking lineage-specific differences in both structure and function have sparked an interest in exploring the molecular evolution of insect USP/RXR, in part to understand the evolution of endocrine regulation, but also to aid in the design of selective pesticides which target the USP/RXR-EcR complex. The use of relative-rate tests revealed high divergence rates in the Lepidoptera and Diptera for both USP/RXR and EcR, particularly in the LBD of USP/RXR [21], [27]. These results suggested that the heterodimer may have coevolved to become functionally divergent in the Holometabola. It was later shown that this event was unique to the Mecopterida, a suborder of insects which includes the Diptera, Trichoptera, Mecoptera, Siphonaptera and Lepidoptera [28], [29]. More recently, this work has been extended to the heterodimer interface between USP/RXR and EcR. By reconstructing and modeling the ancestral Mecopterida heterodimer, Iwema *et al.* [30] demonstrated that an expanded dimerization surface was common to this branch. Furthermore, the data suggested that this enlarged surface was the result of torsion in the structure of USP/RXR caused by the position of loop L1–3 in these insects.

Based on experimental data from *Drosophila*, crystallography work from *Heliothis virescens*, and molecular evolutionary studies, a model for the functional evolution of USP/RXR has been proposed. Iwema *et al.* [21], [30] and Tocchini-Valentini *et al.* [31] suggest the following three groups of USP/RXR: 1. The RXR type which evolved retinoid-binding early, a function retained in the Cnidaria, Mollusca, and Chordata; 2. The Non-Mecopterida type which lost ligand-binding function along an ancient arthropod lineage, a state found in insect orders such as the Hymenoptera, Coleoptera, Orthoptera and Dictyoptera; 3. Finally, the Mecopterida USP type which may have gained a novel ligand-binding function and an enlarged dimerization interface with EcR during the evolution of some higher insects.

To investigate these hypotheses of USP/RXR evolution, we implemented codon-based maximum likelihood methods to estimate the ratio of non-synonymous (*d*_N_) to synonymous (*d*_S_) substitutions across an insect phylogeny and independently along the Mecopterida lineage. This ratio serves as an indicator of selective constraint and such methods have been successfully used to detect positive selection, the signature of functional gain, in a variety of insect gene families and vertebrate nuclear receptors [32], [33], [34], [35], [36]. We also examined among-site variation in *d*_N_/*d*_S_ across a dataset of Mecopterida and Non-Mecopterida insects using random-sites models to investigate changes in site-specific evolutionary rates across key structural and functional motifs. Additionally, several of these analyses were repeated with a dataset of EcR sequences to examine evolutionary constraint in the heterodimer. Finally, we employed newly developed methods which allow the independent estimation of *d*_N_ and *d*_S_ in order to determine the effect among-site variation in *d*_S_ on the patterns of site-specific evolutionary rates observed under random-sites analyses.

## Methods

### Sequence data collection and dataset assembly

USP/RXR and EcR sequences were collected from literature and GenBank using a combination of BLAST and keyword searches (Table S1). Where possible, EcR-A isoform data were used in order to incorporate a larger portion of the protein in subsequent analyses. Both USP/RXR and EcR sequences were not available for all species, and in such cases sequences from the most closely related insects were used instead. Amino acid multiple sequence alignments for USP/RXR and EcR were constructed using ClustalW [37] as implemented in MEGA 4 [38] and adjusted by eye to ensure structural motifs were maintained. Poorly aligned regions and major gaps were deleted (see supporting Figure S1, S2).

To explore selective constraint across the entire phylogeny, both USP/RXR and EcR alignments were truncated such that only the well conserved LBD was included for use in branch and branch-sites analyses (termed USP/RXR LBD and EcR LBD datasets). To examine the variation in evolutionary rates across sites and across functional domains within each group, the USP/RXR and EcR alignments were each split into Mecopterida and Non-Mecopterida datasets (termed USP/RXR A/B-LBD and EcR A/B-LBD datasets for Mecopterida and Non-Mecopterida respectively).

### Estimation of evolutionary rates

In order to test for positive selection and examine changes in site-specific evolutionary rates codon-based likelihood methods were used to estimate *d*_N_/*d*_S_ (ω) ratios across the USP/RXR gene. Under no selective pressure, sequences evolve neutrally, and this is indicated by ω = 1, whereas ω<1 indicates purifying selection, and ω>1 positive selection [39], [40]. To estimate ω several branch-specific [41], [42], branch-site [43], [44], and random-sites [45], [46] models were implemented using the codeml program of the PAML software package (version 4.2b; [47]). Random-sites models which allow for variation in *d*_S_ [48] were also implemented using the HyPhy software package (version 1.0; [49]). In addition, many of these analyses were also carried out for EcR to examine evolutionary constraint in the USP/RXR-EcR heterodimer.

**Figure 1:**
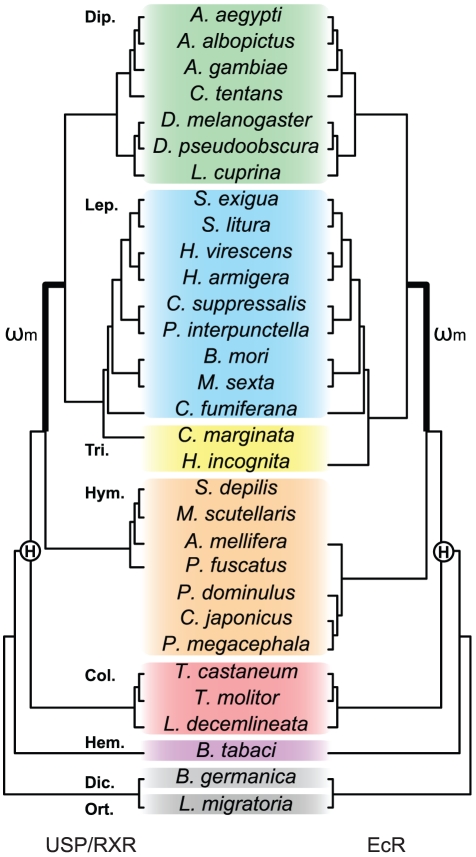
Phylogeny of insect species used in our analysis. The topology of the trees used for the USP/RXR (left) and EcR (right) datasets are based on known taxonomic relationships. Species contained in the Diptera (Dip.) are shaded green, Lepidoptera (Lep.) blue, Trichoptera (Tri.) yellow, Hymenoptera (Hym.) orange, Coleoptera (Col.) red, Hemiptera (Hem.) purple, Dictyoptera (Dic.) and Orthoptera (Ort.) grey. The origin of holometabolous insects is indicated by an encircled bold H. The highlighted branch is the foreground lineage for detecting positive selection, where a separate *d*_N_/*d*_S_ ratio was estimated for the Mecopterida (ω_m_).

The amino acid alignments described above for each of the six datasets were converted into nucleotide data. A tree reflecting current understanding among major insect lineages was used for both USP/RXR and EcR (Figure 1; [50], [51] Insecta; [52] Coleoptera; [53] Lepidoptera; [54] Diptera; [55], [56] Hymenoptera). Gene trees generated with our USP/RXR and EcR alignments, using both maximum-likelihood [57] and neighbor-joining [38], [58] methods, were generally consistent with these known inter-species relationships (see supporting Figure S3). However, low branch support for many of the ordinal relationships resulted in trees with poor resolution of the Non-Mecopterida, therefore all analyses were only performed using the species trees. For analyses carried out on the USP/RXR and EcR A/B-LBD datasets, the trees were modified to include only Mecopterida or Non-Mecopterida taxa. However, this resulted in a tree bifurcated at the root with two clades when only the Mecopterida were considered, so *T. castaneum* was added as an outgroup to form a tripartition tree. Correspondingly, the *T. castaneum* sequence was added to both USP/RXR and EcR A/B-LBD Mecopterida datasets.

It is important to note that our dataset was composed of insect taxa which diverged over 140 million years ago [59], and that the saturation of synonymous substitution rates over such large distances may result in the estimation of *d*_N_/*d*_S_ ratios higher than the actual value [60]. However, this would affect all estimations across all datasets, and our primary objective was to examine relative differences and patterns in substitution rates.

### Branch and branch-site models

To investigate the gain in function hypothesized to have occurred in Mecopterida USP/RXR, branch models were implemented which allowed for an additional ω parameter along the lineage leading to the Mecopterida (Two-ratios), or alternatively along the lineage leading to the ancestor of Mecopterida and Hymenoptera. Codon frequencies were estimated using the F3x4 matrix and models were run from varying starting ω values ranging above and below 1, or κ (relative transition to transversion rate ratio) values ranging from 0 to 5 in order to test for convergence. Likelihood ratio tests (LRTs; [61]) were then conducted to determine statistically significant differences between nested models. Models with the additional ω parameter were compared against M0 where only one ω value was estimated across the phylogeny. Since coevolution of USP/RXR and EcR has been suggested, the above branch models were also applied to the EcR LBD dataset.

Given the elevation in ω identified by branch models, variation in selective pressure among codon sites in Mecopterida USP/RXR was examined using branch-site models applied to the USP/RXR LBD dataset. Branch-site models allow the use of a Bayes Empirical Bayes (BEB) analysis [62] to identify specific positively selected sites within the gene along a given branch. A model with Mecopterida designated as foreground, Model A alt, was compared against a stringent model for positive selection, Model A null, and a less stringent model, M1a. Positive selection is detected if ‘Model A alt’ is a better fit than ‘Model A null’ where classes of positively selected sites have ω set to 1, whereas relaxed purifying selection (possibly suggestive of positive selection) is indicated if ‘Model A alt’ is a better fit than M1a which does not allow a class of sites with ω>1 (referred to as test 2 and test 1 of positive selection by [44]). Sites identified as under positive selection by BEB analysis with a posterior probability (P)>0.95 were subsequently mapped onto the 1.65 Å crystal structure of *H. virescens* USP (PBD 1G2N) or the 2.90 Å crystal structure of the *H. virescens* USP-EcR heterodimer (PBD 1R1K) using the VMD software package [63].

### Random-sites models

In order to compare among-site variation in *d*_N_/*d*_S_ across functional domains of USP/RXR and EcR between Mecopterida and Non-Mecopterida insects, random-sites models were implemented using PAML [45], [46]. Codon frequencies and initial starting values were the same as those used in the above branch and branch-site models. Comparison between M3, with three discrete classes of ω, and null M0 tests for variation in selection pressure among sites, but does not explicitly incorporate positive selection. However, comparisons between both M2a and null M1a (discrete distribution), and M8 and null M7 (where ω is drawn from a beta distribution), test for positive selection by allowing for an additional class of sites with ω>1. Posterior probabilities for site classes under sites models can be calculated either by the Naïve Empirical Bayes (NEB) approach [45], [46] or by the BEB approach [62] which considers sampling errors in the estimation of ω. For models such as M8 where BEB data was available, the posterior mean ω at each site was plotted using site class assignments as calculated by BEB, not NEB.

### Random-sites models that incorporate variation in *d*_S_

To account for the effect of variation of synonymous substitution rates across sites, we implemented several codon selection analyses using the dNdSRateAnalysis program of the HyPhy software package [49]. Similar to M3 in PAML the variable nonsynonymous rates model, or ‘Nonsynonymous’, implemented in HyPhy assumes a constant *d*_S_ (α_S_ = 1), but samples *d*_N_ (β_S_) values from a given rate distribution. However, the dual variable rates model, or ‘Dual’, estimates *d*_S_ (α_S_) and *d*_N_ (β_S_) independently, sampling both from a given rate distribution [48]. In this study, both models were run using the MG94×REV core rate matrix with the GDD (general discrete distribution) rate distribution method using the ‘Independent Discrete’ setting. For each model three synonymous and nonsynonymous rate classes (3×3) were specified. All models were run several times using the randomized initial value option to find the global optimum. To test for site-to-site variation in *d*_S_ across both USP/RXR and EcR A/B-LBD datasets for the Mecopterida and Non-Mecopterida, nested Dual and Nonsynonymous models were compared using LRTs.

## Results

### Branch models

The current theory of USP/RXR functional evolution proposes that the ligand-binding function was lost in an ancient arthropod lineage followed by a subsequent gain in function along the Mecopterida lineage. Additionally, it has been suggested that Mecopterida USP/RXR acquired an expanded dimerization interface with EcR. We tested these hypotheses of functional gain by estimating evolutionary rates across a dataset composed of insect USP/RXR ligand-binding domain sequences (USP/RXR LBD dataset), using codon-based models of substitution (Figure 1). Likelihood scores and ω (*d*_N_/*d*_S_) values, as calculated by PAML, are shown in Table 1. Branch models implemented in PAML allow ω to be freely estimated along specified foreground branches while all other background branches are constrained to the same ω across the phylogeny (Two-ratios model). A branch model analysis, for which the Mecopterida lineage was set as foreground, demonstrated an elevated value (ω_m_ = 0.166) compared to the background (ω = 0.040), but cannot distinguish between relaxed constraint or positive selection at a subset of sites (Table 1). However, the likelihood ratio test (LRT) indicated that the additional parameter did not yield a significantly better fit for the data than the null model M0 where one ω is estimated across the entire phylogeny (p = 0.347).

**Table 1:** Parameter estimates for branch and branch-site models (LBD).

						LRT		
Model	np	*ln*L	κ	Parameter Estimates	Positively Selected Sites	Null	df	p-value
**USP/RXR**								
M0: one-ratio	53	−11110.02	1.537	ω = 0.041	-			
Branch-specific models:								
Two-ratios	54	−11109.58	1.533	ω_0_ = 0.040, ω_1_ = 0.166	-	M0	1	0.347
Branch-site models:								
M1a^a^	54	−11097.30	1.544	ω_0_ = 0.040, ω_1_ = 1.000	-			
				*p*_0_ = 0.990, *p*_1_ = 0.010				
Model A null^b^	55	−11082.55	1.535	ω_0_ = 0.039, ω_1_ = 1.000	-			
				ω_2a_ = 1.000, ω_2b_ = 1.000				
				*p*_0_ = 0.814, *p*_1_ = 0.009				
				*p*_2a_ = 0.175, *p*_2b_ = 0.002				
Model A alt	56	−11079.65	1.536	ω_0_ = 0.040, ω_1_ = 1.000	30 sites: 222, 230, 231,	M1a	2	2.174×10^−08^ *
				ω_2a_ = 8.122, ω_2b_ = 8.122	252, 253,272, 302, 403,	Br-s A null^b^	1	0.016*
				*p*_0_ = 0.796, *p*_1_ = 0.009	411 (at P>0.95)			
				*p*_2a_ = 0.194, *p*_2b_ = 0.002	296, 301, 324, 398			
					(at P>0.99)			
**EcR**								
M0: one-ratio	53	−9701.18	1.661	ω = 0.029				
Branch-specific models:								
Two-ratios	54	−9700.11	1.664	ω_0_ = 0.029, ω_1_ = 0.008		M0	1	0.143

NOTE – np is the number of parameters for each model, df is the degrees of freedom, while *p* is the proportion of sites in a given site class. Sites under positive selection are listed according to the site numbering of the *H. virescens* reference sequence, accession number AX383958.

a Null model for test 1 of positive selection from Zhang *et al.* [44].

b Null model for test 2 from Zhang *et al.* [44], a more stringent test of positive selection.

*Significant (p-value<5%).

Since coevolution of USP/RXR and EcR has been suggested, a branch model for which the Mecopterida lineage was set as foreground was also fit to a dataset composed of EcR ligand-binding domain sequences (EcR LBD dataset). Unlike USP/RXR, the foreground lineage demonstrated a decreased evolutionary rate (ω_m_ = 0.008) suggestive of stronger purifying selection (Table 1). However, the LRT again indicated that the additional parameter did not yield a significantly better fit for the data than M0 (p = 0.143).

In a separate analysis, the lineage leading to the ancestor of Hymenoptera and Mecopterida was also freely estimated across both USP/RXR and EcR LBD sequences to determine if ω was elevated in the branch preceding the Mecopterida. However, in both datasets, ω was found to be below background (ω = 0.002 and ω = 0.0001, respectively; data not shown). The added parameter along that branch also did not yield a statistically better fit than M0 in either dataset (p = 0.072 and p = 0.084, respectively; data not shown). Overall, the branch model results, which only allow for a constant ω parameter across sites, suggest a weak trend where evolutionary rates might be elevated in USP/RXR along the Mecopterida lineage.

### Branch-sites models

The insignificance of the branch models tests may have been a consequence of a lack of positive selection, or positive selection acting only on a few sites within the gene along a given branch. Therefore branch-site models were implemented to estimate ω for each site in the USP/RXR LBD dataset along the specified foreground lineage. Branch-site Model A alt demonstrated that a proportion of sites in the USP/RXR gene have ω>1 in the Mecopterida lineage (Table 1). When compared to both M1a and branch-site Model A null, a more stringent test for positive selection, the LRT showed that the result was statistically significant (p = 2.174×10^−8^ and p = 0.016, respectively; Table 1).

**Figure 2:**
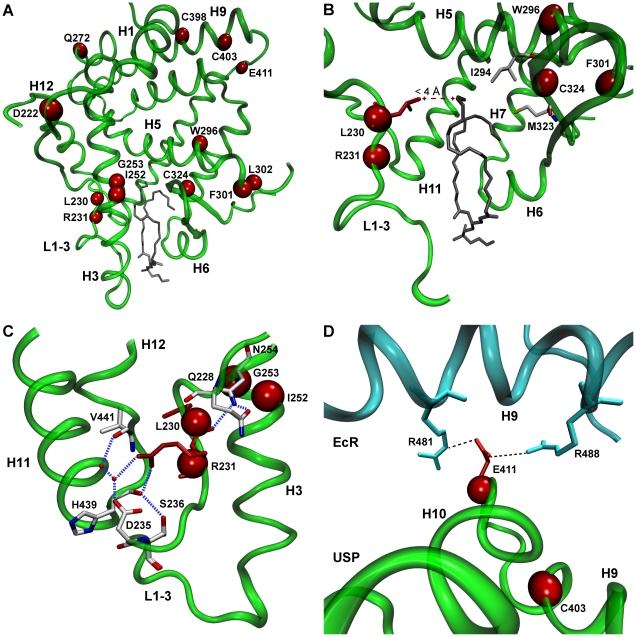
Location of putative positively selected sites in Mecopterida USP/RXR. (A) The distribution of sites inferred by PAML to be under positive selection (P>0.95) along the Mecopterida branch (red spheres) across the crystal structure of *H. virescens* USP (green) with the ligand shown in grey. (B) Positively selected sites (red spheres) located near ligand binding sites (white sidechains). Only L230 directly interacts with the phospholipid ligand (grey) via van der Waals (red dashes). (C) The involvement of sites L203 and R231 in a hydrogen bond network between H3, loop L1–3 and H12. Hydrogen bonds are indicated by dotted blue lines and waters by small red dots (adapted from [24]). The backbone and sidechains of residues not under positive selection are shown in white, with blue and red indicating nitrogen and oxygen respectively. (D) Polar interactions (dashed black lines) between the positively selected site E411 in USP loop L9–10 (green) and two arginine residues in H9 of the EcR (cyan). The images were created using PDB 1G2N and 1R1K, site numbering according to *H. virescens* USP (AX383958) and EcR (Y09009).

Using a Bayes Empirical Bayes (BEB) analysis in Model A alt, a class of sites with ω>1 was identified (Table S2). Nine sites showed ω>1 at a posterior probability (P) 0.99>P>0.95, and four at P>0.99 (Table 1). These sites mapped onto important structural and functional regions of the *H. virescens* USP [24], [25] crystal structure (Figure 2A). Of the 13 sites, one directly interacted with the ligand, and three others lay near ligand-binding sites (Figure 2B). Several sites lay within loop L1–3, and were involved in a hydrogen bond network with loop L11–12 and H3 which stabilizes the position of structural elements in Mecopterida USP/RXR (Figure 2C). Although only one site is known to form direct contact with EcR in the crystal structure of the heterodimer, several others fell within H9, a component of the dimerization core (Figure 2D). One site was located in the coactivator groove in loop L3–4, and two others lay in the region immediately adjacent to loop L5-S1. In addition, several of the sites with P<0.95 also participated in interactions with EcR or were located beside sites which do so (supporting Table S2). Overall, these results indicate significantly elevated ω values along the Mecopterida lineage, indicating positive selection in regions of the protein important for ligand-binding, structural stability and dimerization.

### Random-sites models

**Figure 3:**
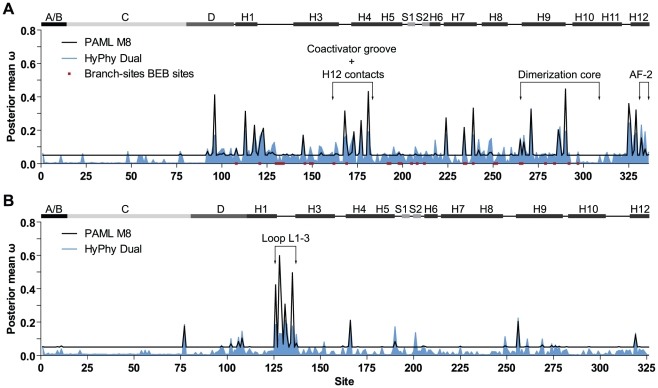
Posterior mean ω at each amino acid site across the USP/RXR gene. The values of ω as estimated by M8 in PAML (black line) and the Dual model of HyPhy (shaded blue) are shown for each codon site across the gene for the Mecopterida (A) and Non-Mecopterida (B). All sites inferred to be under positive selection by branch-site analysis the along the Mecopterida branch are shown as red boxes on the x-axis in of the Mecopterida plot. A schematic of USP/RXR secondary structure is shown above both plots to illustrate the position of each functional domain (A/B, C, and D) as well as the helices (H1–H12) and β sheets (S1–S2) of the ligand-binding domain. The schematic for the Mecopterida and Non-Mecopterida genes are based of the crystal structures of *H. virescens* and *B. tabaci* USP/RXR, respectively. Site numbering is based on the USP/RXR alignment given in supporting figure S1.

In order to compare among-site variation in selection pressure across USP/RXR functional domains between Mecopterida and Non-Mecopterida insects, random-sites models were conducted using PAML with datasets for each group which spanned all domains (USP/RXR A/B-LBD datasets) (Table 2). M3 was the best fit for the Mecopterida and Non-Mecopterida datasets (p = 2.231×10^−127^ and p = 5.063×10^−47^, respectively), suggestive of significant among-site variation in ω. However, M3 is generally a better fit than the null M0 for most proteins as a single ω value across all sites is unlikely. For both the Mecopterida and Non-Mecopterida USP/RXR A/B-LBD datasets, tests of positive selection were not significant. Neither M2a nor M8 were a better fit than the respective null models M1a and M7 (p = 1.0 in both cases). Although no positively selected sites were identified in either Mecopterida or Non-Mecopterida USP/RXR, ω was in fact, elevated at particular regions of the gene (Figure 3A, B). Site class assignments were generally consistent across models, with peaks in ω occurring at the same codon sites, so only the results for M8 are presented (for M3 results see supporting Figure S4). Overall, more sites with elevated ω values were observed in the Mecopterida compared to the Non-Mecopterida.

**Table 2:** Parameter estimates for USP/RXR gene (A/B-LBD).

					LRT		
Model	np	*ln*L	κ	Parameter Estimates	Null	df	p-value
**Mecopterida:**							
M0: one-ratio	35	−10594.82	1.532	ω = 0.035			
M1a: neutral	36	−10531.81	1.622	ω_0_ = 0.029, ω_1_ = 1.000			
				*p*_0_ = 0.950, *p*_1_ = 0.050			
M2a: selection	38	−10531.81	1.622	ω_0_ = 0.029, ω_1_ = 1.000, ω_2_ = 1.000	M1a	2	1.000
				*p*_0_ = 0.950, *p*_1_ = 0.002, *p*_2_ = 0.048			
M3: discrete (*K* = 3)	39	−10297.50	1.564	ω_0_ = 0.002, ω_1_ = 0.046, ω_2_ = 0.194	M0	4	2.231×10^−127^ *
				*p*_0_ = 0.457, *p*_1_ = 0. 450, *p*_2_ = 0.092			
M7: beta	36	−10299.37	1.557	*p* = 0.417, *q* = 9.184			
M8: beta& ω	38	−10299.37	1.557	*p* = 0.417, *q* = 9.185			
				*p*_0_ = 0.99999, (*p*_1_ = 0.00001)	M7	2	1.000
				ω = 1.000			
**Non-Mecopterida:**							
M0: one-ratio	19	−5537.53	1.625	ω = 0.016			
M1a: neutral	20	−5496.97	1.789	ω_0_ = 0.013, ω_1_ = 1.000			
				*p*_0_ = 0.967, *p*_1_ = 0.033			
M2a: selection	22	−5496.97	1.789	ω_0_ = 0.013, ω_1_ = 1.000, ω_2_ = 1.000	M1a	2	1.000
				*p*_0_ = 0.967, *p*_1_ = 0.019, *p*_2_ = 0.014			
M3: discrete (*K* = 3)	23	−5426.21	1.735	ω_0_ = 0.001, ω_1_ = 0.020, ω_2_ = 0.119	M0	4	5.063×10^−47^ *
				*p*_0_ = 0.521, *p*_1_ = 0.394, *p*_2_ = 0.085			
M7: beta	20	−5429.44	1.728	*p* = 0.300, *q* = 13.893			
M8: beta& ω	22	−5429.44	1.728	*p* = 0.300, *q* = 13.895			
				*p*_0_ = 0. 99999, (*p*_1_ = 0. 00001)	M7	2	1.000
				ω = 1.000			

NOTE – np is the number of parameters for each model, df is degrees of freedom, while *p*_0_–*p*_2_ is the proportion of sites in a given site class, and *p*, *q* describe the beta distribution.

*Significant (p-value<5%).

In the Mecopterida, there were a dramatic number of sites with increased ω in the LBD and D domain compared to the A/B and C domains (Figure 3A). These sites clustered within H1, the amino-terminal of loop L1–3, loop L3–4, H4, H7, H9 and H12 of the LBD (see supporting Table S3 for details). Two sites were located beside ligand-binding sites and six other sites lay near residues which make dimerization contacts with EcR. Three sites in H12 lay near residues that interact with loop L1–3, H3 and the coactivator binding groove. Four sites lay in the coactivator binding groove, a region blocked in the Mecopterida as a consequence of contacts with H12 and loop L1–3 [23], [24]. Several of the above sites also overlapped with those identified to be under positive selection along the Mecopterida lineage by branch-site analysis (Figure 3A, red squares). However, many sites differed between these types of analyses as branch-sites models compare the specified foreground branch to the background lineages, whereas random-sites models detect among-site rate variation within a clade. Unlike the Mecopterida, only a handful of sites with an elevated ω were observed in the Non-Mecopterida USP/RXR dataset (Figure 3B; supporting Table S3). These were primarily located in loop L1–3, a region with extensive sequence variation among Non-Mecopterida species [14], [64], [65]. There were also a few sites with elevated ω in H4, H9, H12 and the carboxy-terminal DNA-binding domain (C domain). Overall, among-site variation in the USP/RXR suggests that regions involved in dimerization and the blockage of the coactivator binding groove may be under relaxed constraint within the Mecopterida clade.

**Figure 4:**
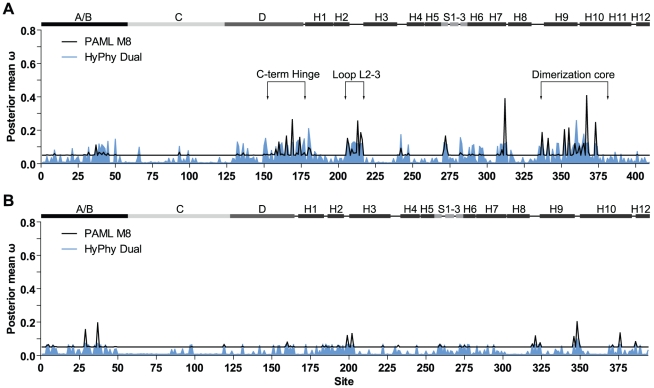
Posterior mean ω at each amino acid site across the EcR gene. The values of ω as estimated by M8 in PAML (black line) and the Dual model of HyPhy (shaded blue) are shown for each codon site across the gene for the Mecopterida (A) and Non-Mecopterida (B). A schematic of EcR secondary structure is shown above both plots to illustrate the position of each functional domain (A/B, C, and D) as well as the helices (H1–H12) and β sheets (S1–S3) of the ligand-binding domain. The schematic for the Mecopterida and Non-Mecopterida genes are based of the crystal structures of *H. virescens* and *B. tabaci* EcR, respectively. Site numbering is based on the EcR alignment given in supporting figure S2.

We also sought to examine among-site variation across EcR functional domains (EcR A/B-LBD datasets), as shifts in USP/RXR structure and function may also have affected the molecular evolution of EcR (Table 3). As with USP/RXR, M3 also yielded the best fit for both Mecopterida and Non-Mecopterida datasets (p = 3.320×10^−97^ and 9.273×10^−32^, respectively). No positively selected sites were identified and neither M2a nor M8 were a better fit than the null models M1a and M7 for either dataset (p = 1.0 in both cases). As in the USP/RXR analyses, *d*_N_/*d*_S_ site-profiles were plotted for EcR based on the M8 result (Figure 4A, B; see supporting Figure S4 for M3 results). Again, more sites with elevated ω values were observed in Mecopterida compared to the Non-Mecopterida.

**Table 3:** Parameter estimates for EcR gene (A/B-LBD).

					LRT		
Model	np	*ln*L	κ	Parameter Estimates	Null	df	p-value
**Mecopterida:**							
M0: one-ratio	35	−10215.15	1.690	ω = 0.029			
M1a: neutral	36	−10198.25	1.737	ω_0_ = 0.028, ω_1_ = 1.000			
				*p*_0_ = 0.988, *p*_1_ = 0.012			
M2a: selection	38	−10198.25	1.737	ω_0_ = 0.028, ω_1_ = 1.000, ω_2_ = 1.000	M1a	2	1.000
				*p*_0_ = 0.988, *p*_1_ = 0.008, *p*_2_ = 0.003			
M3: discrete (*K* = 3)	39	−9987.57	1.721	ω_0_ = 0.000, ω_1_ = 0.023, ω_2_ = 0.107	M0	4	3.320×10^−97^ *
				*p*_0_ = 0.404, *p*_1_ = 0.393, *p*_2_ = 0.203			
M7: beta	36	−9988.17	1.725	*p* = 0.357, *q* = 10.575			
M8: beta& ω	38	−9988.17	1.719	*p* = 0.357, *q* = 10.575			
				*p*_0_ = 0. 99999, (*p*_1_ = 0. 00001)	M7	2	1.000
				ω = 1.000			
**Non-Mecopterida:**							
M0: one-ratio	19	−6357.75	2.022	ω = 0.016			
M1a: neutral	20	−6357.74	2.029	ω_0_ = 0.016, ω_1_ = 1.000			
				*p*_0_ = 0.999, *p*_1_ = 0.001			
M2a: selection	22	−6357.74	2.029	ω_0_ = 0.016, ω_1_ = 1.000, ω_2_ = 1.000	M1a	2	1.000
				*p*_0_ = 0.999, *p*_1_ = 0.001, *p*_2_ = 0.000			
M3: discrete (*K* = 3)	23	−6281.96	2.085	ω_0_ = 0.000, ω_1_ = 0.013, ω_2_ = 0.0737	M0	4	9.273×10^−32^ *
				*p*_0_ = 0.412, *p*_1_ = 0.435, *p*_2_ = 0.153			
M7: beta	20	−6282.43	2.085	*p* = 0.326, *q* = 17.365			
M8: beta& ω	22	−6282.43	1.906	*p* = 0.326, *q* = 17.365			
				*p*_0_ = 0.99999, (*p*_1_ = 0.00001)	M7	2	1.000
				ω = 1.000			

NOTE – np is the number of parameters for each model, df is the degrees of freedom, while *p*_0_–*p*_2_ is the proportion of sites in a given site class, and *p*, *q* describe the beta distribution.

*Significant (p-value<5%).

In the Mecopterida there was a concentration of sites with elevated ω in carboxy-terminal D domain and the dimerization core (Figure 4A; supporting Table S4). Several sites were located in loop L2–3, a region which varies in sequence and is not well modeled in insect crystal structures [21], [25], [26]. One site in the β sheet region was located between several ligand-binding sites. Six sites in H7, H9 and H10 were located near residues which make contact with USP/RXR. Three sites at the amino-terminus of H10 fell into a region associated with dimerization and ligand-binding specificity. Substitutions in this region confer *Aedes aegypti*-like sensitivity to ecdysone to *D. melanogaster* EcR, which is normally only responsive to 20E [66]. As in USP/RXR, there was far less among-site variation in ω across Non-Mecopterida EcR (Figure 4B; supporting Table S4). Three sites in loop L9–10 and H10 align with the aforementioned region in *A. aegypti* which affects sensitivity to ecdysone. In addition, three other sites were located near ligand-binding sites. Overall, these results indicate that the dimerization core of EcR, like that of USP/RXR, may also be under relaxed constraint in the Mecopterida clade.

### Random-sites models that incorporate variation in *d*_S_

Traditional *d*_N_/*d*_S_ methods estimate *d*_N_ and *d*_S_ as a ratio, and assume a constant *d*_S_ [45], [46]. However, this may be an unrealistic assumption for some datasets, and synonymous substitutions are thought to be under selection in flies, nematodes and yeast [67]. Recently, models have been developed to allow for the independent estimation of *d*_N_ and *d*_S_. To examine the effect of among-site variation in *d*_S_ on the estimation of ω in our datasets, we implemented several such models in the HyPhy software package (see supporting Tables S5, S6, S7 for parameter estimates). Overall, the results of HyPhy are similar to those of PAML (Figure 3 and 4, shaded blue). For all datasets the regions with elevated ω were consistent between both methods, with some variation in peak height. The baseline of the *d*_N_/*d*_S_ site-profile plots appears elevated for the PAML M8 results compared to the HyPhy results because the former employs BEB methods, which account for sampling error in the maximum likelihood *d*_N_/*d*_S_ (and proportion) estimates, whereas the latter does not.

For both USP/RXR and EcR A/B-LBD datasets in the Mecopterida site-to-site variation in *d*_S_ was significant when the Dual model compared to the Nonsynonymous null model where *d*_S_ is fixed at all sites (p = 0.037 and 0.025 respectively; Table 4). However, this was not the case with either dataset for the Non-Mecopterida in which significant variation in *d*_S_ among sites was not detected. Given the small size of the Non-Mecopterida datasets and the limited taxonomic sampling, we may lack the statistical power to estimate *d*_N_ and *d*_S_ accurately in this group. Overall the HyPhy results indicate that despite significant among-site variation in *d*_S_ within some datasets, the patterns in substitution rates across both the USP/RXR and EcR A/B-LBD datasets are the same when *d*_N_ and *d*_S_ are estimated independently.

**Table 4:** Synonymous rate variation among sites (HyPhy).

	MG94×REV Nonsynonymous GDD 3		MG94×REV Dual GDD 3×3		LRT	
Dataset	np	Log *L*	np	Log *L*	df	p-value
Mecopterida USP/RXR (A/B-LBD)	43	−10237.10	47	−10232.01	4	0.037*
Non-Mecopterida USP/RXR (A/B-LBD)	27	−5415.51	31	−5410.81	4	0.052
Mecopterida EcR (A/B-LBD)	43	−9946.36	47	−9940.77	4	0.025*
Non-Mecopterida EcR (A/B-LBD)	27	−6269.43	31	−6269.40	4	0.999

NOTE – np is the number of parameters for each model, Log *L* is the logarithm of the maximum likelihood value, and df is the degrees of freedom used in the LRT calculation when comparing Dual versus Nonsynonymous models.

*Significant (p-value<5%).

## Discussion

Our results indicate that USP/RXR is under positive selection along the branch leading to the Mecopterida, with positively selected sites tending to be located in regions involved in ligand-binding, interactions with loop L1–3 and dimerization (Figure 2). Furthermore, random-sites analyses showed that ω was elevated across the Mecopterida clade compared to the Non-Mecopterida for both USP/RXR and EcR (Figure 3, 4). Sites with elevated ω tended to be concentrated in components of the dimerization interface, suggesting relaxed constraint in this region among the Mecopterida (Figure 3A, 4A). There were also several sites with elevated ω in both proteins located near sites important for ligand-binding. Overall, these results are consistent with the acquisition of functional gains with respect to ligand-binding and dimerization in USP/RXR in the Mecopterida.

Although higher relative substitution rates have been shown in the Mecopterida, our study represents the first instance of codon-based likelihood phylogenetic methods being used to estimate *d*_N_/*d*_S_ ratios across the USP/RXR gene [21], [27], [28]. The elevated *d*_N_/*d*_S_ values we observed along the Mecopterida lineage are consistent the with high amino acid substitution rates reported previously [28]. Strikingly, the regions identified as under either positive selection or relaxed constraint play a major role in defining the structure of USP/RXR in the Mecopterida. In order to function as ligand-dependent transcription factors, nuclear receptors must undergo a shift in the position of H12 upon ligand binding to generate an interface for coactivators [68]. Residues in the AF-2 domain and the coactivator binding groove become accessible, allowing the binding of transcriptional coactivators [68], [69], [70]. However, the position of loop L1–3 in Mecopterida USP/RXR prevents shifts in the H12 position, regardless of ligand binding, thereby locking the receptor in an antagonist conformation [23], [24].

These conformational changes are evident in the evolutionary history of USP/RXR. Studies using reconstruction and homology modeling of the ancestral Mecopterida USP/RXR have investigated the origin of an expanded interface with EcR; these novel contacts were a result of intramolecular epistasis whereby the position of loop L1–3 created torsion in the protein, shifting the position of loop L8–9 closer to EcR [30]. We identified sites under positive selection along the Mecopterida lineage in the loop L1–3 region which interact with the ligand and contribute to a hydrogen bond network with H3 and loop L11–12 (Figure 2). Site R385 (according to *H. virescens*) in H9, a Mecopterida-specific contact with EcR, was also shown to be under positive selection along the Mecopterida lineage by branch-site analysis. Furthermore, sites surrounding R385 appear to be under relaxed constraint within the Mecopterida in our random-sites analysis (Figure 3A). This was also the case at sites near S447 (according to *H. virescens*) in EcR H7, which establishes contact with USP/RXR R385 (Figure 4A). This is consistent with the acquisition of an expanded dimer interface between USP/RXR and EcR in the Mecopterida.

It has been postulated that the expansion of this interface may reflect a strengthened inter-dependency between USP/RXR and EcR for activation [30]. Based on structural studies, Diptera and Lepidoptera USP/RXR cannot bind the canonical NR-box LXXLL motif of coactivators because H12 sits within the binding site [23], [24], [71], [72]. Our random-sites analyses revealed sites with elevated ω within both H12 and the coactivator binding groove (loop L3–4, H4) of Mecopterida USP/RXR (Figure 3A). These results are consistent with a change in the function of these regions, leading to a relaxation of evolutionary constraint at these sites. Without a novel interaction interface, such shifts may render USP/RXR more dependent on partner proteins for coactivator recruitment [23], [73]. Interestingly, similar shifts in cofactor interfaces may also have occurred in EcR. An elevation in ω was observed in the carboxy-terminal D domain of Mecopterida EcR, a region implicated in corepressor binding in the mammalian thyroid hormone receptor [74]. The biological significance of an increased dependence on partner proteins remains unclear, but there are some possibilities.

One mechanism which may have led to increased evolutionary rates in the Mecopterida dimer interface is the increased recruitment of USP/RXR as a protein hub. In *D. melanogaster* and *A. aegypti*, USP/RXR is known to interact with many other proteins such as Seven-up (Svp), hormone receptors 38 and 78 (HR38, 78), as well as the *Methoprene-tolerant* gene product (MET) [75], [76], [77], [78]. Hubs at major branch points in protein-protein interaction networks tend to display evidence of elevated ω rates and positive selection [79]. The strong positive selection we observed could be congruent with such a role for USP/RXR. Indeed network level analyses suggest that several proteins in the ecdysone cascade, including USP/RXR and EcR, experienced an increased evolutionary rate at the base of the Mecopterida [80]. However, it is unclear if this is due to functional divergence or simply parallel accelerations across the network in order to maintain interactions. Similarly, the elevated evolutionary rates we detected within the dimerization interface of the Mecopterida clade may be the result of a combination of strong positive selection and purifying selection, not simply relaxed constraint. Substitutions in this region may affect both the affinity and specificity of protein-protein interaction, but might not necessarily improve both of these attributes. In fact, there may be some trade-offs, whereby some substitutions confer a tighter affinity for one protein, but reduce the specificity of interactions with other partner proteins. However, at this point the residues involved in the interface between USP/RXR and alternate partner proteins remain unknown. Furthermore, the network of USP/RXR protein interactions is unknown in lower insects, and a lack of sequence data from outside of the Holometabola prevented the analysis of alternate USP/RXR partners in our study. Therefore, further work is needed to clarify the protein-protein interactions of USP/RXR among insect lineages.

The link between shifts in USP/RXR molecular evolution and shifts in physiology at the organismal level has been difficult to establish. Studies have shown that the role of USP/RXR in molting and metamorphosis seems conserved across insect lineages [6], [7]. Differences in the reproductive biology of insect lineages have been largely unexplored, but may hold more promise [28]. Although there are some exceptions, JH is the major hormone regulating reproductive events such as vitellogenesis in most insect groups [81]. In the Lepidoptera, there is a decreased dependence on JH for vitellogenesis, whereas in the Diptera, vitellogenesis is largely regulated by ecdysteroids. In many Diptera, JH is instead required during previtellogenesis in order for tissues to acquire competence to 20E [82]. The site of vitellogenin production also varies between insect lineages. In many basal lineages, the fat body appears to be the sole site of synthesis, in others, both the ovary and fat body contribute, whereas in some Diptera, the ovary may be the major source [83], [84]. Thus, a stronger relationship between USP/RXR and EcR for heterodimerization is also consistent with the increased dependence on ecdysteroids for the regulation of reproductive events in some higher insects.

USP/RXR is involved in many reproductive events in higher insects such as egg chamber formation and chorion gene expression in *Drosophila* and regulation of the cyclicity of vitellogenesis in *A. aegypti* [75], [85], [86]. Ovarian morphology and structure also varies among insects groups, particularly in lineages between the basal Non-Mecopterida and Mecopterida, such as the Paraneoptera, Coleoptera and Neuropterida [87], [88]. The genes which control reproductive events differ among ovary types as well. For example, the set of genes responsible for chorion formation in meroistic ovaries differs from those in the panoistic type ovary, found in the basal Non-Mecopterida [87], [89]. In addition, USP/RXR and EcR are highly expressed in nurse cells, a cell type unique to the meroistic ovary [85], [90]. Thus, shifts in ovarian morphology, development and the endocrine control of reproduction may have also required plasticity in nuclear receptors. However, the role of USP/RXR in the reproductive events of basal insects is not known, as is its role in highly derived insects contained within the Mecopterida.

Finally, it is important to consider caveats of *d*_N_/*d*_S_ based methods. In recent years a debate has emerged regarding the use of these methods to reliably detect positive selection because of potential false positives identified in some genes [91], [92], [93]. However, in our dataset the positively selected sites identified along the Mecopterida lineage by branch-site analysis were consistent with the structural and experimental work of other researchers. Furthermore, we used patterns of *d*_N_/*d*_S_ variation across sites, not merely positive selection, to draw conclusion regarding USP/RXR evolution. Finally, we utilised new models which allow for among-site variation in *d*_S_ and observed strikingly similar patterns in site-specific evolutionary rates even across these different methods of estimating *d*_N_/*d*_S_.

While computational methods, such as those used here, are useful in identifying patterns of selective constraint acting on USP/RXR across insect lineages, the functional changes generated by the substitutions identified here remain speculative. For example, we cannot clarify whether or not sites under positive selection near the ligand-binding pocket are related to the potential acquisition of JH binding. Further experimental evidence is required to understand the physiological implications of these evolutionary shifts. In particular, examining the role of USP/RXR in the reproductive system of more basal insects, for example, with the use of techniques such as RNAi, may shed light on these issues. Additionally, the apparent increase in the rigidity of Mecopterida USP/RXR makes this an excellent system for the use molecular dynamics studies to investigate subtle shifts in structure. Future work in this area may yield more insights into the role of the positively selected sites identified in this study which impact the conformation of the receptor complex. Overall, our results highlight the need for more comparative physiological work in the basal Non-Mecopterida and provide critical site-specific information for the design of future site directed mutagenesis studies on USP/RXR.

## Supporting Information

Figure S1 **Alignment of insect USP/RXR sequences.** The green and orange bars indicate the Mecopterida and Non-Mecopterida taxa, respectively, and the schematic below the alignment shows the USP/RXR domain structure. For ease of viewing one alignment is shown. However, the complete alignment was not used for analysis as some regions do not align (e.g. D domain). Only the carboxy-terminal E/F (*), or ligand-binding domain, was used for the branch and branch-sites analyses reported in table 1. Arrows indicate where poorly aligned regions and major gaps were deleted. Full length sequences were used for the random-sites and HyPhy analyses where the larger dataset was split into Mecopterida and Non-Mecopterida only datasets in order to compare evolutionary rates between the two groups. For clarity, site numbering for the full length Mecopterida (green) and Non-Mecopterida (orange) datasets is shown above and below the alignment, respectively. Note that species names have been abbreviated to six characters, complete names can be found in supporting table S1.
(PDF)

Figure S2 **Alignment of insect EcR sequences.** The green and orange bars indicate the Mecopterida and Non-Mecopterida taxa, respectively, and the schematic below the alignment shows the EcR domain structure. For ease of viewing one alignment is shown. However, the complete alignment was not used for analysis as some regions do not align (e.g. D domain). Only the carboxy-terminal E (*), or ligand-binding domain, was used for the branch analyses reported in table 1. Arrows indicate where poorly aligned regions and major gaps were deleted. Full length sequences were used for the random-sites and HyPhy analyses where the larger dataset was split into Mecopterida and Non-Mecopterida only datasets in order to compare evolutionary rates between the two groups. For clarity, site numbering for the full length Mecopterida (green) and Non-Mecopterida (orange) datasets is shown above and below the alignment, respectively. Note that species names have been abbreviated to six characters, complete names can be found in supporting table S1.
(PDF)

Figure S3 **USP/RXR and EcR gene trees.** Gene trees for USP/RXR and EcR were generated using the alignment of LBD sequences given in supporting figures S1 and S2. Maximum-likelihood trees for USP/RXR (A) and EcR (C) were constructed in PhyML [57] using the WAG substitution model, with four rate categories to estimate the gamma parameter shape. Neighbor-joining [58] trees for USP/RXR (B) and EcR (D) were constructed in MEGA 4 [38] using the Poisson correction model, with the pair-wise deletion of gaps. For all analyses 100 bootstrap replicates were performed, and nodes with values less than 60 were later collapsed. Each tree was then rooted along the branch leading to *B. germanica* and *L. migratoria*. Note that species names have been abbreviated, see supporting table S1.
(PDF)

Figure S4 ***d*** **_N_/** ***d*** **_S_ site-profile plots for PAML model M3.** The values of ω as estimated by M3 in PAML using the NEB method are shown for each codon site across Mecopterida USP/RXR (A), Non-Mecopterida USP/RXR (B), Mecopterida EcR (C) and Non-Mecopterida EcR (D). A schematic of USP/RXR and EcR secondary structure is shown above each plot to illustrate the position of each functional domain (A/B, C, and D) as well as the helices (H1–H12) and β sheets of the ligand-binding domain. These schematics are based on the crystal structure of each gene in *H. virescens* and *B. tabaci*. Site numbering is the same as figures 3 and 4.
(PDF)

Table S1 **Sequence data information.**
(DOC)

Table S2 **Putative positively selected sites.**
(DOC)

Table S3 **Sites with elevated ω in the USP/RXR gene.**
(DOC)

Table S4 **Sites with elevated ω in the EcR gene.**
(DOC)

Table S5 **Statistics for rate distributions inferred by HyPhy.**
(DOC)

Table S6 **Rate class assignment for MG94×REV Nonsynonymous GDD 3.**
(DOC)

Table S7 **Rate class assignment for MG94×REV Dual GDD 3×3.**
(DOC)
