# Supplementary figures and images for: Molecular Evolution of Ultraspiracle Protein (USP/RXR) in Insects
Source: PLoS One. 2011 Aug 25;6(8):e23416. doi: 10.1371/journal.pone.0023416 (PMC3162005; doi:10.1371/journal.pone.0023416)

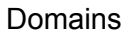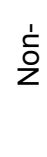

Supplement: Figure S1 — Alignment of insect USP/RXR sequences. The green and orange bars indicate the Mecopterida and Non-Mecopterida taxa, respectively, and the schematic below the alignment shows the USP/RXR domain structure. For ease of viewing one alignment is shown. However, the complete alignment was not used for analysis as some regions do not align (e.g. D domain). Only the carboxy-terminal E/F (*), or ligand-binding domain, was used for the branch and branch-sites analyses reported in table 1. Arrows indicate where poorly aligned regions and major gaps were deleted. Full length sequences were used for the random-sites and HyPhy analyses where the larger dataset was split into Mecopterida and Non-Mecopterida only datasets in order to compare evolutionary rates between the two groups. For clarity, site numbering for the full length Mecopterida (green) and Non-Mecopterida (orange) datasets is shown above and below the alignment, respectively. Note that species names have been abbreviated to six characters, complete names can be found in supporting table S1. (PDF) [file pone.0023416.s001.pdf]

**A**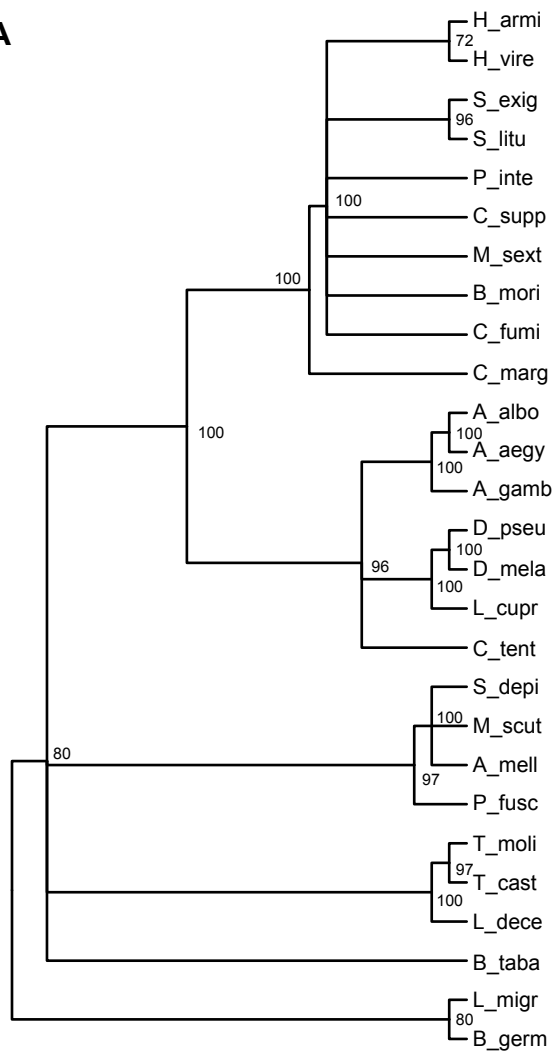**B**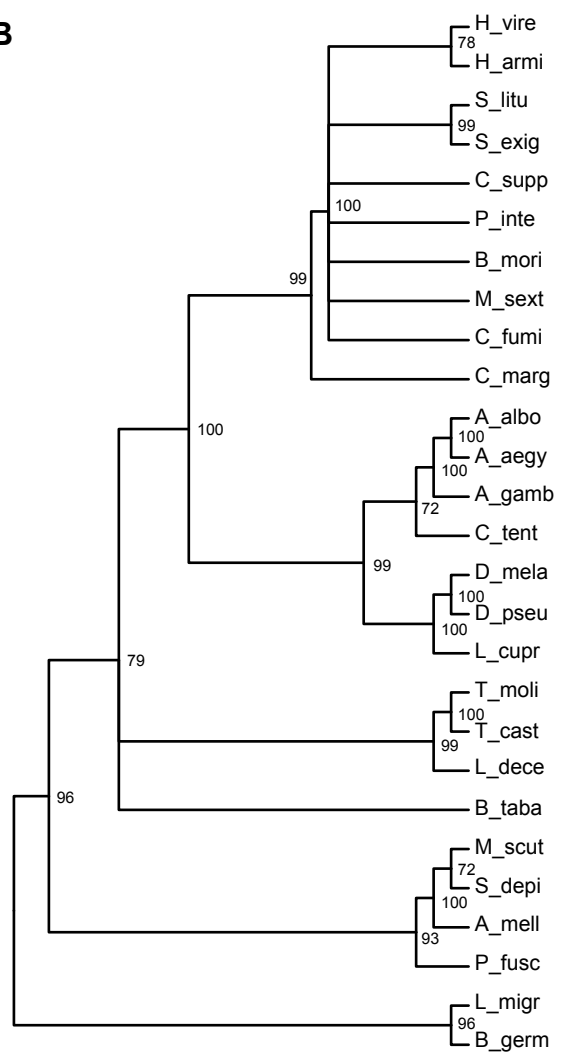**C**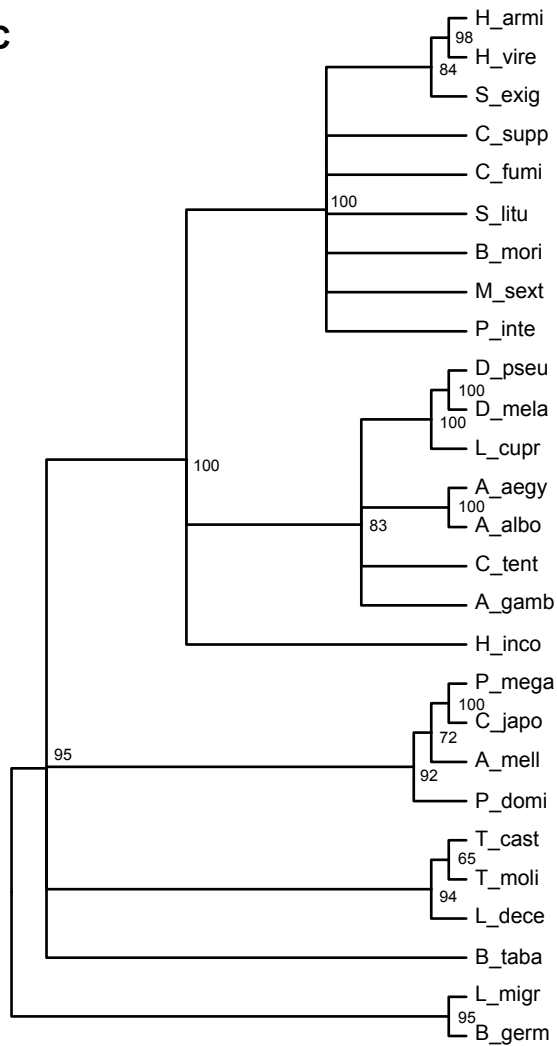**D**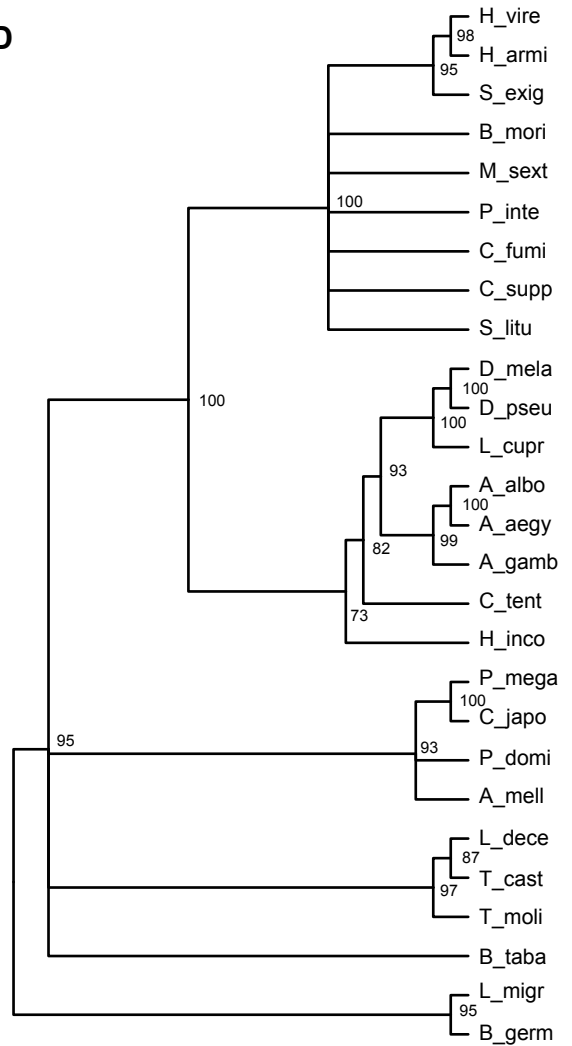

Supplement: Figure S3 — USP/RXR and EcR gene trees. Gene trees for USP/RXR and EcR were generated using the alignment of LBD sequences given in supporting figures S1 and S2. Maximum-likelihood trees for USP/RXR (A) and EcR (C) were constructed in PhyML [57] using the WAG substitution model, with four rate categories to estimate the gamma parameter shape. Neighbor-joining [58] trees for USP/RXR (B) and EcR (D) were constructed in MEGA 4 [38] using the Poisson correction model, with the pair-wise deletion of gaps. For all analyses 100 bootstrap replicates were performed, and nodes with values less than 60 were later collapsed. Each tree was then rooted along the branch leading to B. germanica and L. migratoria. Note that species names have been abbreviated, see supporting table S1. (PDF) [file pone.0023416.s003.pdf]

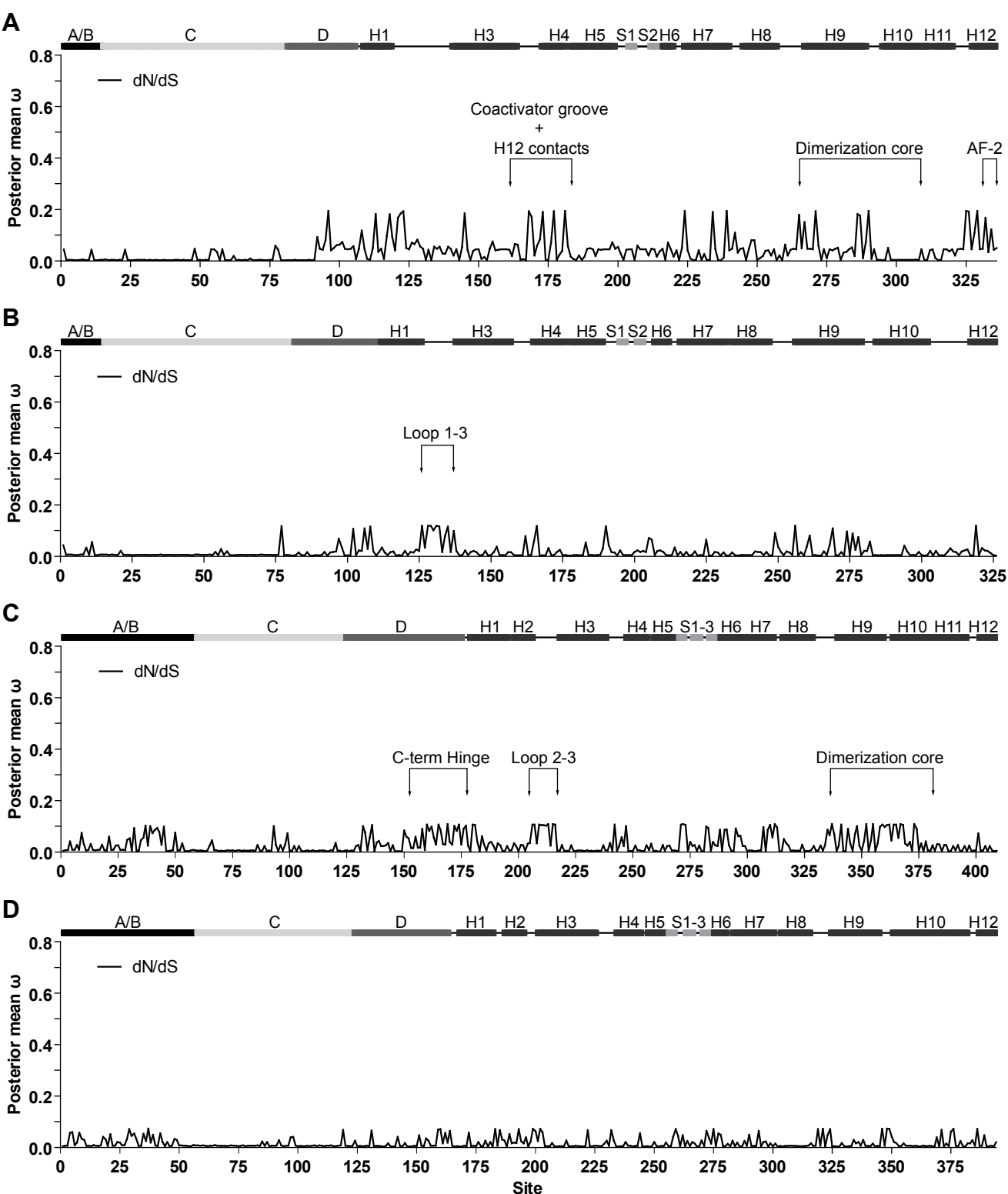

Supplement: Figure S4 — d N/ d S site-profile plots for PAML model M3. The values of ω as estimated by M3 in PAML using the NEB method are shown for each codon site across Mecopterida USP/RXR (A), Non-Mecopterida USP/RXR (B), Mecopterida EcR (C) and Non-Mecopterida EcR (D). A schematic of USP/RXR and EcR secondary structure is shown above each plot to illustrate the position of each functional domain (A/B, C, and D) as well as the helices (H1–H12) and β sheets of the ligand-binding domain. These schematics are based on the crystal structure of each gene in H. virescens and B. tabaci. Site numbering is the same as figures 3 and 4. (PDF) [file pone.0023416.s004.pdf]
