# Supplementary material for: Molecular Evolution of Ultraspiracle Protein (USP/RXR) in Insects
Source: PLoS One. 2011 Aug 25;6(8):e23416. doi: 10.1371/journal.pone.0023416 (PMC3162005; doi:10.1371/journal.pone.0023416)
Supplement: Table S1 — Sequence data information. (DOC) [file pone.0023416.s005.doc]

**Table S1. Sequence data information.**

|  |  | **USP/RXR** | **EcR** |
| --- | --- | --- | --- |
| **Group** | **Species** | **Accession No.** | **Accession No.** |
| **Dictyoptera** | *Blattella germanica* | AJ854489 | AM039690 |
| **Orthoptera** | *Locusta migratoria* | AY348873 | AF049136 |
| **Hemiptera** | *Bemisia tabaci* | EF174330 | EF174329 |
| **Coleoptera** | *Tenebrio molitor* | AJ251542 | Y11533 |
|  | *Leptinotarsa decemlineata* | AB211193 | AB211191 |
|  | *Tribolium castaneum* | AM295014 | AM295015 |
| **Hymenoptera** | *Scaptotrigona depilis* | DQ190542 |  |
|  | *Melipona scutellaris* | AY840093 |  |
|  | *Polistes fuscatus* | AY827156 |  |
|  | *Polistes dominulus* |  | DQ083517 |
|  | *Apis mellifera* | AY273778 | AB490017 |
|  | *Pheidole megacephala* |  | AB194765 |
|  | *Camponotus japonicus* |  | AB296080 |
| **Diptera** | *Lucilia cuprina* | AY007213 | U75355 |
|  | *Drosophila melanogaster* | X53417 | NM_165461 |
|  | *Drosophila pseudoobscura* | XM_001355316 | XM_002138935 |
|  | *Aedes albopictus* | AF210734 | AF210733 |
|  | *Aedes aegypti* | AF305213 | AY345989 |
|  | *Chironomus tentans* | AF045891 | S60739 |
|  | *Anopheles gambiae* | XM_320944 | XM_320323 |
| **Trichoptera** | *Chimarra marginata* | DQ083513 |  |
|  | *Hydropsyche incognita* |  | DQ083516 |
| **Lepidoptera** | *Chilo suppressalis* | AB081840 | AB067811 |
|  | *Bombyx mori* | U06073 | D87118 |
|  | *Heliothis virescens* | AX383958 | Y09009 |
|  | *Choristoneura fumiferana* | AF016368 | AF092030 |
|  | *Manduca sexta* | U44837 | U49246 |
|  | *Plodia interpunctella* | AY619987 | AY489269 |
|  | *Spodoptera exigua* | EU642475 | EU426551 |
|  | *Spodoptera litura* | EU180022 | EU180021 |
|  | *Helicoverpa armigera* | EU526832 | EU526831 |
