# Supplementary material for: Molecular Evolution of Ultraspiracle Protein (USP/RXR) in Insects
Source: PLoS One. 2011 Aug 25;6(8):e23416. doi: 10.1371/journal.pone.0023416 (PMC3162005; doi:10.1371/journal.pone.0023416)
Supplement: Table S2 — Putative positively selected sites. (DOC) [file pone.0023416.s006.doc]

**Table S2. Putative positively selected sites.**

| **Site in** | **Posterior** | **Region** | **Function in the Mecopterida^c^** |
| --- | --- | --- | --- |
| **HvUSP^a^** | **Probability^b^** |  |  |
| 209 | 0.609 | H1 |  |
| 222 | 0.967* | L1-3 |  |
| 229 | 0.921 | L1-3 | Beside ligand-binding site  van der Waals contacts with H12  H-bond network with H3, H11-H12 in *Drosophila* |
| 230 | 0.973* | L1-3 | Ligand-binding site (van der Waals)  H-bond network with H3, H11-H12  van der Waals contacts with H3, L11-12 in *Drosophila* |
| 231 | 0.989* | L1-3 | Beside ligand-binding site  H-bond network with H3, H11-H12  van der Waals contacts with H3 in *Drosophila* |
| 232 | 0.606 | L1-3 | H-bond network with H3, H11-H12  van der Waals contacts with H3, L11-12 in *Drosophila* |
| 233 | 0.927 | L1-3 | H-bond network with H3 |
| 249 | 0.632 | H3 | Ligand-binding site (van der Waals) |
| 252 | 0.970* | H3 | Near sites in H-bond network with H3, H11-H12 |
| 253 | 0.968* | H3 | Ligand-binding site in *Drosophila* (non-polar)  Near sites in H-bond network with H3, H11-H12 |
| 265 | 0.911 | H3 | Part of coactivator groove where H12 lies |
| 272 | 0.987* | L3-4 | Within coactivator groove region |
| 295 | 0.726 | H5 | Beside ligand-binding site |
| 296 | 0.999** | H5 | Beside site in H-bond network with H5, H3, β-sheet |
| 301 | 0.995** | H5 |  |
| 302 | 0.954* | L5-S1 |  |
| 324 | 0.994** | S1 | Beside ligand-binding site  Beside site in H-bond network with H5, H3, β-sheet |
| 327 | 0.717 | LS1-S2 | Near ligand-binding sites in *Drosophila* |
| 331 | 0.572 | S2 | Ligand-binding site in *Drosophila* (non-polar) |
| 353 | 0.885 | H7 |  |
| 354 | 0.738 | H7 | Beside dimerization site |
| 358 | 0.824 | H7 |  |
| 370 | 0.644 | H8 |  |
| 371 | 0.664 | H8 |  |
| 384 | 0.692 | L8-9 | Beside dimerization site |
| 385 | 0.871 | H9 | Mecopterida-specific dimerization site |
| 398 | 0.996** | H9 | Within dimerization core |
| 403 | 0.985* | H9 | Within dimerization core |
| 411 | 0.953* | L9-10 | Polar interactions with EcR sites R481 and R488 |
| 416 | 0.535 | H10 | Near several dimerization sites |

*P>0.95, **P>0.99

^a^ Site numbers according to *H. virescens* reference sequence, accession number AX383958.

^b^ Probability that ω>1, as determined by BEB analysis

^c^ Data according to Billas *et al.* [24], Iwema *et al.* [30] and Clayton *et al.* [23].
