# Supplementary material for: Molecular Evolution of Ultraspiracle Protein (USP/RXR) in Insects
Source: PLoS One. 2011 Aug 25;6(8):e23416. doi: 10.1371/journal.pone.0023416 (PMC3162005; doi:10.1371/journal.pone.0023416)
Supplement: Table S3 — Sites with elevated ω in the USP/RXR gene. (DOC) [file pone.0023416.s007.doc]

**Table S3. Sites with elevated ω in the USP/RXR gene.**

| **Site in** | **Site in** | **ω^b^** | **Region** | **Function^c^** |
| --- | --- | --- | --- | --- |
| **ref seq^a^** | **dataset** |  |  |  |
| **Mecopterida** | |  |  |  |
| 194 | 96 | 0.411 | D | Poorly aligned region |
| 214 | 113 | 0.313 | H1 |  |
| 219 | 118 | 0.229 | H1 |  |
| 222 | 121 | 0.126 | L1-3 |  |
| 223 | 122 | 0.181 | L1-3 |  |
| 224 | 123 | 0.212 | L1-3 | Near sites which interact with H3 |
| 248 | 145 | 0.168 | H3 | Beside ligand-binding site |
| 271 | 168 | 0.315 | L3-4 | Within coactivator groove region |
| 272 | 169 | 0.129 | L3-4 | Within coactivator groove region |
| 276 | 173 | 0.190 | H4 | Within coactivator groove region, interacts with H12 |
| 280 | 177 | 0.258 | H4 | Within coactivator groove region  Beside site that interacts with H12 |
| 284 | 181 | 0.431 | L4-5 | Beside site that interacts with H12 |
| 343 | 224 | 0.274 | H7 | Near ligand-binding site, and several in *Drosophila* |
| 353 | 234 | 0.216 | H7 | Near dimerization site |
| 358 | 239 | 0.332 | H7 | Near dimerization site |
| 384 | 265 | 0.142 | L8-9 | Beside dimerization site |
| 386 | 267 | 0.128 | H9 | Beside dimerization site |
| 390 | 271 | 0.324 | H9 | Near dimerization site |
| 405 | 286 | 0.202 | H9 | Within dimerization core |
| 406 | 287 | 0.144 | H9 | Within dimerization core |
| 409 | 290 | 0.446 | L9-10 | Near dimerization site |
| 444 | 325 | 0.359 | L11-12 | Near sites which interact with L1-3 and H3 |
| 445 | 326 | 0.255 | H12 | Near sites which interact with L1-3 and H3 |
| 448 | 329 | 0.321 | H12 | Near sites in *Drosophila* which interact with L1-3  Near sites which interact with coactivator groove |
| 451 | 332 | 0.153 | AF-2 | Beside site which interacts with coactivator groove |
| **Non-Mecopterida** | |  |  |  |
| - | 77 | 0.175 | C |  |
| 28 | 108 | 0.103 | D |  |
| 46 | 126 | 0.423 | L1-3 | Highly variable sequence, alt. splicing in some taxa |
| 48 | 128 | 0.598 | L1-3 | Same as above |
| 49 | 129 | 0.329 | L1-3 | Same as above |
| 51 | 131 | 0.307 | L1-3 | Same as above |
| - | 132 | 0.128 | L1-3 | Same as above |
| 54 | 135 | 0.495 | L1-3 | Same as above |
| 85 | 166 | 0.211 | H4 |  |
| 176 | 256 | 0.204 | H9 | Within dimerization core |
| 239 | 319 | 0.124 | H12 |  |

^a^ Site numbers according to *H. virescens* (AX383958) and *B. tabaci* (EF174330) reference sequences for the Mecopterida and Non-Mecopterida, respectively.

^b^ As determined by BEB analysis in PAML random-sites model M8; sites listed here have an ω value greater than twice the baseline ω.

^c^ Data according to Billas *et al.* [24], Iwema *et al.* [30] and Clayton *et al.* [23].
