# Supplementary material for: Molecular Evolution of Ultraspiracle Protein (USP/RXR) in Insects
Source: PLoS One. 2011 Aug 25;6(8):e23416. doi: 10.1371/journal.pone.0023416 (PMC3162005; doi:10.1371/journal.pone.0023416)
Supplement: Table S4 — Sites with elevated ω in the EcR gene. (DOC) [file pone.0023416.s008.doc]

**Table S4. Sites with elevated ω in the EcR gene.**

| **Site in** | **Site in** | **ω^b^** | **Region** | **Function^c^** |
| --- | --- | --- | --- | --- |
| **ref seq^a^** | **dataset** |  |  |  |
| **Mecopterida** | |  |  |  |
| 143 | 37 | 0.110 | A/B |  |
| 296 | 160 | 0.128 | D | May bind co-repressors in vertebrates |
| 301 | 165 | 0.165 | D | Same as above |
| 305 | 169 | 0.264 | D | Same as above |
| 310 | 174 | 0.157 | Pre H1 |  |
| 342 | 206 | 0.151 | L2-3 |  |
| 344 | 207 | 0.113 | L2-3 |  |
| 350 | 213 | 0.255 | L2-3 |  |
| 352 | 215 | 0.185 | L2-3 |  |
| 353 | 216 | 0.144 | L2-3 |  |
| 409 | 272 | 0.165 | LS1-S2 | Near sites which affect LBP shape and ligand-binding |
| 449 | 312 | 0.389 | L7-8 | Near dimerization sites |
| 474 | 337 | 0.186 | L8-9 | Near dimerization sites |
| 478 | 341 | 0.151 | H9 | Beside dimerization site |
| 489 | 352 | 0.203 | H9 | Beside dimerization site |
| 492 | 355 | 0.214 | H9 |  |
| 496 | 359 | 0.120 | L9-10 |  |
| 499 | 362 | 0.104 | H10 | Affects sensitivity to ecdysone in *A. aegypti* |
| 502 | 365 | 0.121 | H10 | Affects sensitivity to ecdysone in *A. aegypti* |
| 504 | 367 | 0.408 | H10 | Beside dimerization site  Beside site critical to ecdysone sensitivity in *A. aegypti* |
| 510 | 373 | 0.246 | H10 | Between dimerization sites |
| **Non-Mecopterida** | |  |  |  |
| - | 29 | 0.155 | A/B |  |
| - | 37 | 0.196 | A/B |  |
| 100 | 199 | 0.119 | L2-3 | Upstream of ligand-binding sites |
| 103 | 202 | 0.131 | H3 | Upstream of ligand-binding sites |
| 222 | 321 | 0.117 | L8-9 | Near dimerization sites |
| 247 | 346 | 0.113 | L9-10 | Near *A. aegypti* sites that affect sensitivity to ecdysone |
| 249 | 348 | 0.203 | L9-10 | Affects sensitivity to ecdysone in *A. aegypti* |
| 250 | 349 | 0.103 | H10 | Near *A. aegypti* sites that affect sensitivity to ecdysone |
| 277 | 376 | 0.135 | H10 | Beside ligand-binding site  Downstream of sites which affect LBP shape |

^a^ Site numbers according to *H. virescens* (Y09009) and *B. tabaci* (EF174329) reference sequences for the Mecopterida and Non-Mecopterida, respectively.

^b^ As determined by BEB analysis in PAML random-sites model M8; sites listed here have an ω value greater than twice the baseline ω.

^c^ Data according to Billas *et al.* [25], Hörlein *et al.* [72], Iwema *et al.* [30], Carmichael *et al.* [26] and Wang *et al.* [64].
