# Supplementary material for: Molecular Evolution of Ultraspiracle Protein (USP/RXR) in Insects
Source: PLoS One. 2011 Aug 25;6(8):e23416. doi: 10.1371/journal.pone.0023416 (PMC3162005; doi:10.1371/journal.pone.0023416)
Supplement: Table S5 — Statistics for rate distributions inferred by HyPhy. (DOC) [file pone.0023416.s009.doc]

**Table S5. Statistics for rate distributions inferred by HyPhy.**

| **Dataset** | **CV(α_s_)** | **E(β_s_)** | **CV(β_s_)** | **E(β_s_/ α_s_)** | **CV(β_s_/ α_s_)** |
| --- | --- | --- | --- | --- | --- |
| **MG94 x REV Nonsynonymous GDD 3** |  |  |  |  |  |
| Mecopterida USP/RXR (A/B-LBD) | N/A | 0.046 | 1.270 | 0.046 | 1.270 |
| Non-Mecopterida USP/RXR (A/B-LBD) | N/A | 0.023 | 1.827 | 0.023 | 1.827 |
| Mecopterida EcR (A/B-LBD) | N/A | 0.036 | 1.308 | 0.036 | 1.308 |
| Non-Mecopterida EcR (A/B-LBD) | N/A | 0.038 | 9.558 | 0.038 | 9.558 |
| **MG94 x REV Dual GDD 3 x 3** |  |  |  |  |  |
| Mecopterida USP/RXR (A/B-LBD) | 0.302 | 0.042 | 1.264 | 0.049 | 1.467 |
| Non-Mecopterida USP/RXR (A/B-LBD) | 0.741 | 0.017 | 1.855 | 0.025 | 2.660 |
| Mecopterida EcR (A/B-LBD) | 0.192 | 0.035 | 1.305 | 0.039 | 1.581 |
| Non-Mecopterida EcR (A/B-LBD) | 1.267 | 0.046 | 9.574 | 0.060 | 9.749 |

NOTE – CV is the coefficient of variation, E the expected value, N/A not applicable, α_s_ the synonymous substitution rate, and β_s_ the nonsynonymous substation rate.
