# Supplementary material for: Molecular Evolution of Ultraspiracle Protein (USP/RXR) in Insects
Source: PLoS One. 2011 Aug 25;6(8):e23416. doi: 10.1371/journal.pone.0023416 (PMC3162005; doi:10.1371/journal.pone.0023416)
Supplement: Table S6 — Rate class assignment for MG94×REV Nonsynonymous GDD 3. (DOC) [file pone.0023416.s010.doc]

**Table S6. Rate class assignment for MG94 x REV Nonsynonymous GDD 3.**

| **Dataset** | **Rates Class** | **Rate** | **Sites in Class** |
| --- | --- | --- | --- |
| Mecopterida USP/RXR (A/B-LBD) | 1 | 0.002 | 156 |
|  | 2 | 0.051 | 131 |
|  | 3 | 0.197 | 49 |
| Total sites: |  |  | 336 |
| Non-Mecopterida USP/RXR (A/B-LBD) | 1 | 0.002 | 198 |
|  | 2 | 0.033 | 102 |
|  | 3 | 0.191 | 26 |
| Total sites: |  |  | 326 |
| Mecopterida EcR (A/B-LBD) | 1 | 0.000 | 212 |
|  | 2 | 0.028 | 111 |
|  | 3 | 0.128 | 86 |
| Total sites: |  |  | 409 |
| Non-Mecopterida EcR (A/B-LBD) | 1 | 0.004 | 263 |
|  | 2 | 0.064 | 131 |
|  | 3 | 7.339 | 0 |
| Total sites: |  |  | 394 |

NOTE – The Nonsynonymous model assumes α_s_=1, thus given rates are equivalent to β_s_ or β_s_/ α_s_.
