# Supplementary material for: Molecular Evolution of Ultraspiracle Protein (USP/RXR) in Insects
Source: PLoS One. 2011 Aug 25;6(8):e23416. doi: 10.1371/journal.pone.0023416 (PMC3162005; doi:10.1371/journal.pone.0023416)
Supplement: Table S7 — Rate class assignment for MG94×REV Dual GDD 3×3. (DOC) [file pone.0023416.s011.doc]

**Table S7. Rate class assignment for MG94 x REV Dual GDD 3 x 3.**

| **Dataset** | **Rates** | **β=0.002** | **β=0.047** | **β=0.182** | **Row sum** |
| --- | --- | --- | --- | --- | --- |
| Mecopterida USP/RXR (A/B-LBD) | α=0.407 | 48 | 45 | 14 | 107 |
|  | α=0.946 | 45 | 29 | 15 | 89 |
|  | α=1.401 | 62 | 56 | 22 | 140 |
| Column sum: |  | 155 | 130 | 51 | 336 |

| **Dataset** | **Rates** | **β=0.002** | **β=0.024** | **β=0.141** | **Row sum** |
| --- | --- | --- | --- | --- | --- |
| Non-Mecopterida USP/RXR (A/B-LBD) | α=0.088 | 33 | 14 | 4 | 51 |
|  | α=0.675 | 107 | 58 | 13 | 178 |
|  | α=2.618 | 58 | 31 | 8 | 97 |
| Column sum: |  | 198 | 103 | 25 | 326 |

| **Dataset** | **Rates** | **β=0.000** | **β=0.027** | **β=0.124** | **Row sum** |
| --- | --- | --- | --- | --- | --- |
| Mecopterida EcR (A/B-LBD) | α=0.298 | 43 | 15 | 11 | 69 |
|  | α=0.635 | 40 | 28 | 23 | 91 |
|  | α=1.053 | 129 | 66 | 54 | 249 |
| Column sum: |  | 212 | 109 | 88 | 409 |

| **Dataset** | **Rates** | **β=0.003** | **β=0.049** | **β=6.306** | **Row sum** |
| --- | --- | --- | --- | --- | --- |
| Non-Mecopterida EcR (A/B-LBD) | α=0.728 | 163 | 76 | 0 | 239 |
|  | α=2.130 | 44 | 32 | 0 | 76 |
|  | α=6.905 | 56 | 23 | 0 | 79 |
| Column sum: |  | 263 | 131 | 0 | 394 |

NOTE – Counts of sites are given for each synonymous (α) row x nonsynonymous (β) column in a given dataset.
