# Supplementary material for: Molecular Evolution of Ultraspiracle Protein (USP/RXR) in Insects
Source: PLoS One. 2011 Aug 25;6(8):e23416. doi: 10.1371/journal.pone.0023416 (PMC3162005; doi:10.1371/journal.pone.0023416)
Supplement: Figure S2 — Alignment of insect EcR sequences. The green and orange bars indicate the Mecopterida and Non-Mecopterida taxa, respectively, and the schematic below the alignment shows the EcR domain structure. For ease of viewing one alignment is shown. However, the complete alignment was not used for analysis as some regions do not align (e.g. D domain). Only the carboxy-terminal E (*), or ligand-binding domain, was used for the branch analyses reported in table 1. Arrows indicate where poorly aligned regions and major gaps were deleted. Full length sequences were used for the random-sites and HyPhy analyses where the larger dataset was split into Mecopterida and Non-Mecopterida only datasets in order to compare evolutionary rates between the two groups. For clarity, site numbering for the full length Mecopterida (green) and Non-Mecopterida (orange) datasets is shown above and below the alignment, respectively. Note that species names have been abbreviated to six characters, complete names can be found in supporting table S1. (PDF) [file pone.0023416.s002.pdf]

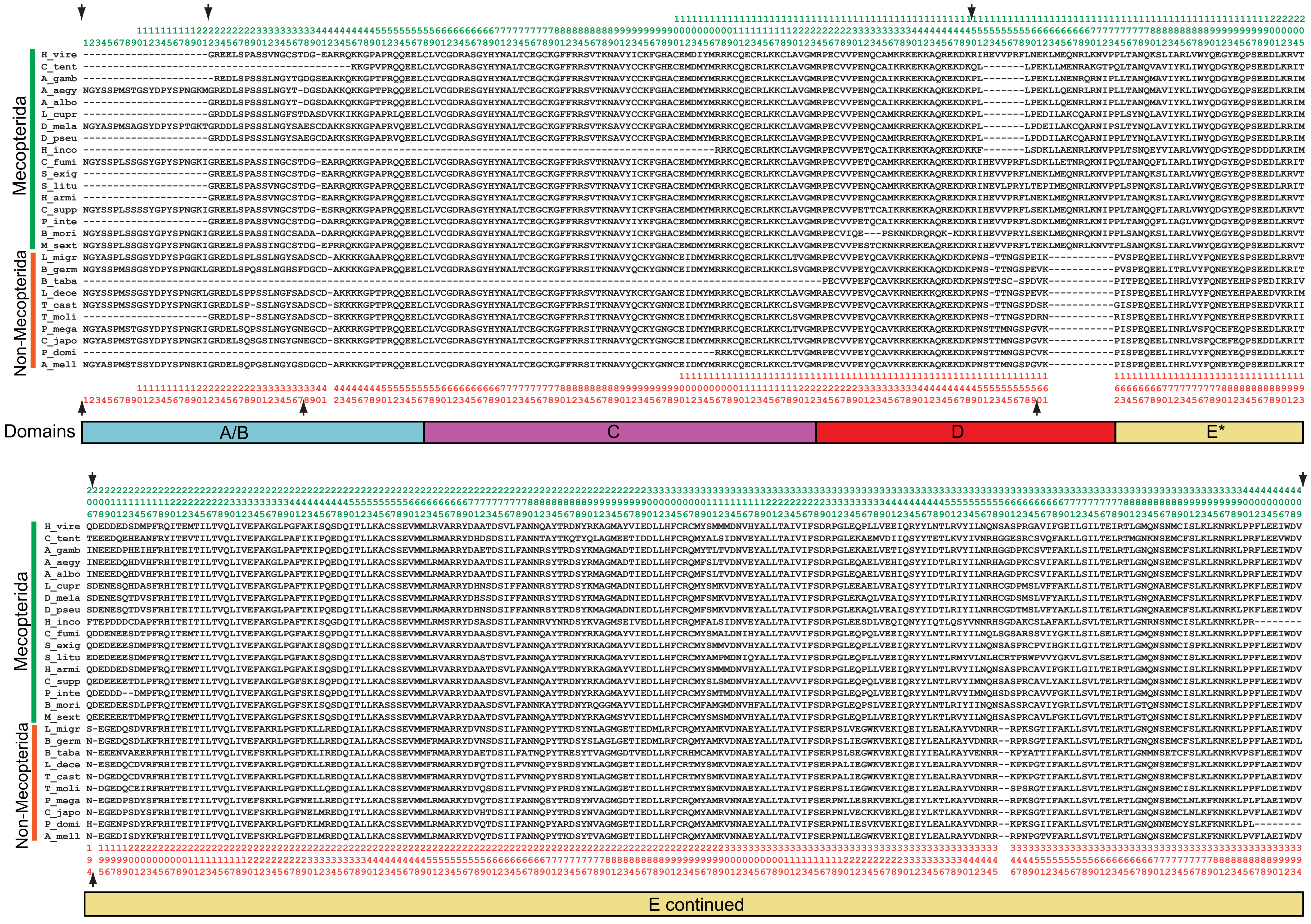

Mecopterida
Non-Mecopterida
H_vire
C_tent
A_gamb
A_aegy
A_albo
L_cupr
D_mela
D_pseu
H_inco
C_fumi
S_exig
S_litu
H_armi
C_supp
P_inte
B_mori
M_sext
L_migr
B_germ
B_taba
L_dece
T_cast
T_moli
P_mega
C_japo
P_domi
A_mell
Domains
A/B
C
D
E*
E continued
